# Inflammation Regulates Haematopoietic Stem Cells and Their Niche

**DOI:** 10.3390/ijms23031125

**Published:** 2022-01-20

**Authors:** Nicole Pui-Yu Ho, Hitoshi Takizawa

**Affiliations:** 1 Laboratory of Stem Cell Stress, International Research Center for Medical Sciences (IRCMS), Kumamoto University, Kumamoto 860-0811, Japan; nicole-h@kumamoto-u.ac.jp; 2 Center for Metabolic Regulation of Healthy Aging (CMHA), Kumamoto University, Kumamoto 860-0811, Japan

**Keywords:** inflammation, haematopoietic stem cells, bone marrow niche

## Abstract

Haematopoietic stem cells (HSCs) reside in the bone marrow and are supported by the specialised microenvironment, a niche to maintain HSC quiescence. To deal with haematopoietic equilibrium disrupted during inflammation, HSCs are activated from quiescence directly and indirectly to generate more mature immune cells, especially the myeloid lineage cells. In the process of proliferation and differentiation, HSCs gradually lose their self-renewal potential. The extensive inflammation might cause HSC exhaustion/senescence and malignant transformation. Here, we summarise the current understanding of how HSC functions are maintained, damaged, or exhausted during acute, prolonged, and pathological inflammatory conditions. We also highlight the inflammation-altered HSC niche and its impact on escalating the insults on HSCs.

## 1. Introduction

Haematopoiesis is a continuous process to produce all lineages of blood and immune cells. Such a lifelong regenerative capacity is maintained by a very minor population of hematopoietic stem cells (HSCs) at the top of the hierarchy. HSCs possess high self-renewal potential with a very slow turnover and undergo stepwise multi-lineage differentiation to give rise to lineage-committed haematopoietic progenitor cells (HPCs) as well as various haemato-immune cells. Compared with HSCs, HPCs have limited self-renewal capacity but higher proliferation potential to presumably maintain daily haematopoiesis. This stepwise haematopoietic hierarchy is advantageous, especially under proliferation stress by ensuring a high degree of flexibility to blood cell differentiation and, at the same time, reduces the considerable proliferation/differentiation burden on HSCs solely. Adult HSCs reside in bone marrow (BM) and are supplied with pivotal factors by a specialised microenvironment, termed the HSC niche. The HSC niche provides immunosuppression and minimises excessive immune reactions in BM so that the primitive HSCs can be protected from immune attacks [1,2,3]. This is also why BM is considered an immune-privileged organ. However, when the haematopoietic system is insulted by exogenous stimuli such as inflammation, infection, or tissue damage, it can be rapidly reshaped to generate a large number of immune effector cells to eliminate the harm. With the increased cell cycling for differentiation and self-renewal, HSCs gradually accumulate DNA damages and lose their long-term reconstitution potential [4].

Inflammatory stress originates from diverse sources. Bacterial or viral infection exemplifies non-sterile inflammation that frequently occurs and highlights how the host responds to external stimuli. Mild local infection can be quickly cleared by innate immune cells such as neutrophils and tissue-resident macrophages. Severe infection may elicit exaggerated immune responses and trigger systemic inflammatory response syndrome (sepsis). Sterile inflammation refers to the other inflammatory responses triggered by non-microbial sources such as injured or dying cells, which release pro-inflammatory cytokines or immunogenic antigens. Imbalanced metabolism may result in excessive redox responses that generate highly reactive chemicals such as reactive oxygen species (ROS) and nitrogen oxide (NO) and trigger immune responses. Environmental sources such as UV or radiation exposure can promote oxidative stress on the skin, gut, and haematopoietic stem cells. Intake of caffeine and alcohol, obesity, chronic diseases, and ageing are also important factors to promote inflammation.

In this review, we discuss how HSCs respond to acute and prolonged inflammatory stress, and how the metabolic changes during inflammation influence the HSCs. Finally, we highlight how the stress-induced remodelling of the HSC niche collaboratively affects the HSC function.

## 2. Inflammatory Processes

**Figure 1:**
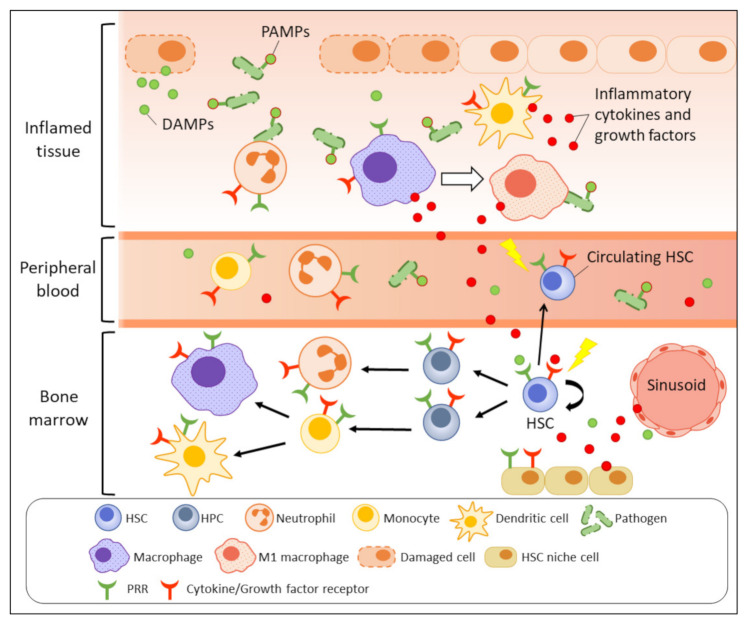
Emergency myelopoiesis triggered by inflammatory signals. Upon infection or tissue damage, the release of PAMPs and DAMPs (green dots) at inflammatory site are recognised by PRRs expressed on immune cells such as macrophages and dendritic cells, as well as the primitive HSCs (direct activation). The activated antigen-presenting cells massively produce inflammatory molecules (red dots) to recruit neutrophils and macrophages to eradicate the pathogens or dead cells. The secreted inflammatory cytokines and growth factors also indirectly activate BM and peripheral HSPCs through respective receptors to increase their mobilisation and myelopoiesis. The stimulated HSC niche under inflammation also contributes to accumulation of inflammatory molecules in BM and increases inflammatory stress on BM HSPCs.

Inflammation refers to a series of highly organised, physiological, and immunological responses that are triggered by stimulatory signals released from pathogens (non-sterile), damaged cells (sterile), or allergens. Inflammation is a part of our body’s protective mechanism and serves the purpose of eliminating the origins of the harmful insults and initiating tissue repair and regeneration. In contrast to adaptive immunity, innate immunity is considered the frontline mechanism driving the inflammation process, which is triggered by pathogen sensing as the initial step (Figure 1). Innate immune cells, especially tissue-resident macrophages and dendritic cells, can recognise the invading pathogens, or more specifically, their pathogen-associated molecular patterns (PAMPs), which are sets of conserved microbial molecules shared within a class of microbe, by respective pattern-recognition receptors (PRRs) expressed on the immune cell surface. Toll-like receptors (TLRs) and nucleotide oligomerisation domain (NOD)-like receptors (NLRs) are well-studied PRRs for pathogen sensing. Damage-associated molecular patterns (DAMPs) that are derived from damaged or dying cells also initiate similar inflammatory responses by activating the corresponding PRRs. Following the pathogen sensing, the activated innate immune cells massively produce a variety of pro-inflammatory chemokines, cytokines, and growth factors, including interleukin (IL)-1, IL-6, tumour necrosis factor (TNF)-α, and interferon (IFN)-γ to recruit more immune effectors, e.g., neutrophils, monocytes, and macrophages to the inflammatory site [5]. Neutrophils quickly eliminate pathogens by phagocytosis, degranulation, or neutrophil extracellular traps [6,7,8]. The cytokines also drive macrophages towards M1 polarisation to support the pathogen killing by enhanced microbicidal activity [9,10]. Meanwhile, the activated antigen-presenting cells, e.g., dendritic cells link the innate and adaptive immunity by presenting the antigens to T cells in form of major histocompatibility complex (MHC) class II to promote T-cell activation and differentiation [11,12]. During the resolution phase, macrophages are induced towards anti-inflammatory M2 polarisation and promote tissue repair by producing growth factors, for instance, platelet-derived growth factor (PDGF), transforming growth factor (TGF)-β1, insulin-like growth factor (IGF)-1, and vascular endothelial growth factor (VEGF)-α to support cell proliferation and angiogenesis [9,10,13].

## 3. Acute Inflammation and HSPCs

The increasing demand for replenishment of short-lived myeloid cells during inflammation exerts a massive amount of proliferative stress on haematopoietic system and triggers enhanced de novo generation of myeloid cells in a process termed “emergency myelopoiesis (granulopoiesis)” [14,15]. To initiate the characteristic haematopoietic responses, haematopoietic stem and progenitor cells (HSPCs) are activated via direct or indirect mechanisms. Apart from the mature immune cells, HPCs and even the quiescent HSCs express abundant TLRs, particularly TLR2 and TLR4, to allow their direct activation and skewed differentiation towards myelopoiesis by microbial signals [16,17,18]. The indirect HSPC activation is mainly mediated by the pro-inflammatory molecules secreted by the activated innate immune cells. Upon TLR ligation, HSPCs can also act as immune effectors to secrete Th2 cytokines, such as IL-5, IL-13, IL-6, and granulocyte-macrophage colony-stimulating factor (GM-CSF), in IL-3- and stem cell factor (SCF)-dependent manner [17,19,20,21]. Apart from the haematopoietic cells, BM niche cells such as endothelial cells, osteoblasts, and mesenchymal stem cells (MSCs) in the HSC niche also abundantly express TLRs and cytokine receptors and can quickly respond to inflammatory signals [22,23,24,25,26,27,28]. How the inflammation-induced disrupted homeostasis in BM niche subsequently brings adverse effects on the fitness of HSPCs will be discussed in a later section. Collectively, HSPCs can be activated directly through TLRs or indirectly through cytokine/growth factor receptors to mediate their haematopoietic responses.

***HSC Expansion and Differentiation:*** many pro-inflammatory cytokines and growth factors, for instance, granulocyte colony-stimulating factor (G-CSF), macrophage colony-stimulating factor (M-CSF), GM-CSF, IL-3, IL-6, TNF-α, IFNs, and Fms-related receptor tyrosine kinase 3 (Flt3) ligands, have been demonstrated to bias HSPC differentiation towards myelopoiesis at the expense of lymphopoiesis [29,30]. Some of the molecules, such as TNF-α, IL-1, and M-CSF are more potent in promoting HSC expansion [31,32,33,34,35,36,37]. Repetitive direct TLR4 activation by LPS infections has been shown to drive quiescent HSCs to cell cycling and diminish their self-renewal capacity [18,38]. However, considering that indirect cytokine stimulation from the surrounding cells may collaboratively affect the HSC function, more comprehensive and careful studies will be necessary to demonstrate that sole TLR activation on HSPCs can alter their behaviours independently. IFN-γ, one of the critical cytokines controlling inflammation, seems to have dual and opposing effects on regulating HSC proliferation, possibly in a context-dependent manner. IFN-γ was shown to activate HSC proliferation during infections [31,39,40]; conflicting findings have indicated that IFN-γ can impair HSC regeneration by limiting their self-renewal instead of altering their quiescence or cell cycling [40,41,42,43]. It is speculated that the discrepancy stems from the indirect influence from the HSC niche and the involvement of stem cells antigen-1 (Sca-1) as an HSC marker [41]. Despite its debatable effect on HSC proliferation, there is consensus that IFN-γ causes functional declines in HSCs [39,41].

***HSC Apoptosis and Senescence:*** inflammation-enhanced proliferation may accumulate DNA damages in HSCs, driving the stem cells to apoptosis or senescence. IFN-γ has been demonstrated to induce HSC apoptosis by activating pro-apoptotic PI3K signalling [44,45]. However, recent studies showed opposing evidence that IFN-γ can drive HSC proliferation with no elevation in apoptosis [32,39]. While the IFN-γ level may be one of the factors determining the apoptosis rate, it is suggested that the IFN-γ-driven apoptosis can only be triggered in the presence of other cytokines [32]. By contrast, TNF-α, another key pro-inflammatory cytokine, is cytotoxic to most of the haematopoietic populations, except the primitive HSCs [29]. TNF-α protects HSCs from necrosis by activating NF-κB pathways to improve their survival during inflammation [29,46]. To our best knowledge, there is, thus far, no direct evidence indicating that infection can induce HSC senescence. Nevertheless, that telomere shortening is the factor known to determine HSC senescence, and serial transplantations that induce massive cytokine storms can significantly reduce the telomere lengths in HSCs [47,48]. This implies that inflammation-induced HSC proliferation and regeneration may promote the occurrence of HSC senescence.

***HSC Mobilisation:*** inflammation induces HSCs to migrate from BM to bloodstream upon activation of NOD1, TLR2, and TLR4 [49,50,51,52]. A part of the mobilised HSCs transfer to the spleen for extramedullary haematopoiesis [49]. G-CSF has been demonstrated to pose potent stimulatory effects on HSC mobilisation without altering their proliferation and has been routinely used for clinical transplantation as a mobilising regimen [49,53,54]. The attenuation of HSC supportive cytokines and adhesion molecules such as stromal cell-derived factor-1 (SDF-1/CXCL12) and vascular cell adhesion molecule 1 (VCAM-1) secreted by the HSC niche cells is another major mechanism of the HSC mobilisation [17,51,55]. Intriguingly, the HSCs that remain in BM during infection undergo highly migratory movements to move away from the original niche [56]. This suggests that inflammation can disrupt the original stem cell–niche interaction and force the HSCs to mobilise, to find a more supportive microenvironment to restore their fitness.

## 4. Metabolic Changes upon Inflammation

In a steady state, HSCs rely primarily on anaerobic glycolysis over oxidative phosphorylation (OXPHOS) for energy generation, albeit the less efficient ATP production, compared with the TCA cycle and electron transport chain [57]. This is likely because HSCs are located in the relatively hypoxic microenvironment inside BM, in which oxygen content is low (<32mmHg pO_2_ in murine BM) despite high vascular density [57]. Compared with other BM cells, HSCs have particularly lower oxygen consumption, higher glycolytic rate, and lower mitochondrial potential [58]. Hypoxia-inducible factor 1-α (HIF-1α) is also more stabilised in HSCs over HPCs, and this is not dependent on the hypoxic microenvironment but the tight transcriptional regulation of HIF-1α in HSCs [58,59,60]. Therefore, the intracellular hypoxia in HSCs is a strategy to maintain their stemness rather than an adaptation to the environment. HSCs with depletion of HIF-1α are more susceptible to cell cycling and ROS generation under 5-FU-induced proliferative stress [59]. Increasing the HIF-1α by removing its negative regulator Cited2 impairs HSC quiescence and reconstitution capacity and subjects the HSCs to apoptosis [61]. Strikingly, the importance of HIF-1α in maintaining HSC quiescence has been recently challenged by new data that HIF-1α deficiency implies no effects on HSC self-renewal, multilineage reconstitution capacity, and recovery from repetitive 5-FU-induced haematopoietic injuries [62]. The discrepancy may be attributed to the simultaneous HIF-1α deletion in non-hematopoietic HSC niche cell populations in the former report, which, in turn, synergised the effect of HIF-1α deletion on HSC quiescence [63].

In the context of inflammation, the energy metabolism in HSCs is reprogrammed from anaerobic glycolysis to oxidative respiration [64,65]. Lowered electron transport chain in HSCs promotes their multi-lineage blood reconstitution by inducing autophagy to control differentiation and self-renewal [66]. This implies that the higher mitochondrial activity in HSCs during inflammation may lead to their exhaustion or functional declines. Moreover, ROS are generated as by-products in the electron transfer reactions. The harmful effects of ROS on HSC quiescence have been well documented [67,68]. Dampening CXCR4/CXCL12 signalling in HSCs increases intracellular oxidative stress and promotes DNA damage responses to resolve the damages caused by the increased mitochondrial ROS production [69]. Accumulated oxidative stress was also shown to drive HSC senescence and/or apoptosis by excessive DNA damages [68].

Apart from HSCs, inflammation also reprograms the metabolism of HSC niche cells. Similar to HSCs, MSCs normally rely on glycolysis to maintain their stem cell potential. The switch from glycolysis to OXPHOS can activate MSC proliferation and impair their quiescence [70,71]. Inflammatory signals from TNFα and IFNγ can stimulate mitochondrial ROS production in MSCs and trigger HIF-1α-mediated MSC immunosuppressive functions [3]. Pro-inflammatory cytokine IL-17 induces ROS generation from MSCs via activation of TNF receptor-associated factor 6 (TRAF6) and Act1 to stimulate proliferation, migration, and osteoblastic differentiation [72,73]. ROS also mediates mitochondrial transfer from MSCs to HSCs to stimulate bioenergetic changes that favours emergency granulopoiesis [64]. In osteoarthritis, sustained elevation of IL-1β level shifts the metabolism of BM chondrocytes to increase glycolysis and, in turn, promote lactate dehydrogenase A (LDHA)-mediated ROS generation [74]. Therefore, each compartment in BM contributes to the accumulation of oxidative stress during inflammation and finally harms the quiescence of HSCs when the ROS level in BM reaches a threshold.

## 5. Chronic Inflammation

Despite the immunosuppressive BM microenvironment, prolonged inflammatory stimuli induce changes in functions and behaviours of mature immune cells and the primitive HSCs. Sustained microbial infection with *Mycobacterium avium* elevates the IFNγ level and irreversibly impairs haematopoiesis by depleting HSCs and suppressing the myelopoiesis [39,75]. Low-grade chronic inflammation developed during the ageing process is recognised as “inflammageing” and is associated with the degradation of HSC fitness over time [76]. In the following section, we review the impacts of prolonged inflammatory conditions on HSCs in various chronic diseases.

***Obesity:*** it is well recognised that obesity has a close correlation with increased systemic inflammation and is considered a low-grade inflammatory disease because adipocyte is a rich source of inflammatory factors [77]. High-fat diet-induced obesity increases marrow adiposity and numbers of granulocytes, monocytes, and haematopoietic progenitors in murine BM [78,79]. However, the extent of HSC expansion varies in different diet-induced obesity models probably due to the varying duration and different percentage of fat in the high-fat diet feeding [79]. The adipocyte accumulation in BM adversely affects HSCs by impairing haematopoietic reconstitution potentials, increasing myelopoiesis, and suppressing lymphopoiesis [79,80,81]. Meanwhile, the preferential differentiation of BM MSCs to adipocyte lineage impairs osteogenesis and bone regeneration, making the HSC niche less supportive to maintain the HSCs [80].

***Atherosclerosis:*** in recent years, emerging reports revealed the relationship between obesity, cardiovascular diseases, and haematopoiesis. Atherosclerosis, a chronic inflammatory coronary artery disease, is characterised by the accumulation of inflammatory lipoproteins and leukocytes such as monocytes, macrophages, and neutrophils that cause atherosclerotic lesions. [82]. Homeostasis of cholesterol is important to maintain primitive BM HSCs. Adenosine triphosphate-binding cassette (ABC) transporters ABCA1 and ABCG1 play pivotal roles in controlling cholesterol efflux from macrophage foam cells and allow high-density lipoprotein (HDL, also considered as “good” cholesterol), and apolipoprotein A1 (ApoA-1) to remove the excessive cholesterol molecules. HDL is well known to be associated with reducing the incidence of atherosclerosis and coronary heart disease. Depletion of ABCA1 and ABCG1 can worsen atherosclerosis by increasing leucocytosis and monocytosis and can induce expansion of HSPCs with enhanced cell cycling. *Abca1*^−/−^ *Abcg1*^−/−^ promote TLR/Myd88-independent inflammatory responses, and elevating HDL levels can inhibit the proliferation of HSPCs and myeloid progenitors by promoting cholesterol efflux [83]. Similarly, dysregulated cholesterol level by removing apolipoprotein E (ApoE), which also promotes cholesterol efflux, on the HSPC surface leads to HSC expansion especially after a high-cholesterol Western-type diet [84]. Dysregulation of cholesterol also adversely impacts the supportive functions of non-haematopoietic cells to HSCs in the HSC niche. Macrophages deficient in ABCA1 and ABCG1 show higher TLR4 expression and stronger inflammatory responses by cholesterol accumulation in membranes [85]. Deficiency of ABCA1 and ABCG1 in macrophages and dendritic cells promotes G-CSF-dependent HSC mobilisation and extramedullary haematopoiesis by increasing plasma IL-17 and splenic IL-23 levels [86]. The reduced expression of HSC maintenance gene CXCL12 in N-Cadherin^+^ osteoblasts and Nestin^+^ MSCs in *Abca1^−/−^ Abcg1^−/−^* mice may impair the HSC quiescence in BM [86]. Recent studies have reported that exercise can reduce HSC proliferation and mobilisation to the periphery by increasing CXCL12 production from LepR^+^ stromal cells [87]. This is probably due to the reduced visceral adipose tissue after exercise to suppress the level of leptin, an adipocytokine from fat tissues, in blood and BM [87]. Exercise can also enhance immunity by improving emergency haematopoiesis during LPS infection and sepsis [87].

***Other inflammation-associated/autoimmune diseases:*** type 2 diabetes, another obesity-related chronic disease, is related to abnormal haematopoiesis as well. Enhanced proliferation of HSCs has been found in mouse models of diet-induced diabetes due to less CXCL12 secretion by BM endothelial cells [88]. Meanwhile, the endothelial cells counteract by increasing epithelial growth factor receptor (EGFR) signalling to regulate HSPC retention via angiopoietin-1 in diabetes [88]. In ulcerative colitis, the enhanced serum levels of IFN-γ in patients are highly associated with the development of clonal haematopoiesis, especially with *DNMT3A* and *PPM1D* mutations [89]. The systemic increase in IL-1 and TNF in rheumatoid arthritis promotes myeloid-biased differentiation in HSCs, along with broad suppression on diverse HSC-niche cell types [90,91]

***Hematologic malignancies*:** continuous dysregulated inflammatory stress on HSCs may contribute to the emergence of mutations and confer selective advantages to certain HSC clones, promoting clonal haematopoiesis. Functional loss of TET2, an epigenetic regulator, is a common mutation in myeloid malignancies and promotes haematopoietic transformation by elevating the self-renewal capacity of the mutated cells [92,93]. Under TNF-α-induced inflammatory stress, *Tet2*-deficient or *Tet2*-mutant human HSCs show stronger proliferative advantage and resistance to apoptosis over the wild-type counterparts [94]. This indicates that the acquisition of genetic ablations in clonal haematopoiesis can subsequently promote the emergence of pre-leukaemic stem cells, followed by myeloid malignancies. Intriguingly, prolonged inflammation promotes the emergence of leukaemic stem cells, but at the same time, the activation of inflammatory signalling may impair the self-renewal of leukaemic stem cells by increasing their cycling and differentiation in acute myeloid leukaemia (AML) [93]. Apart from the intrinsic factor, inflammatory MSCs also induce genotoxic stress on HSCs as extrinsic factors. Dysregulated MSCs in a mouse model of pre-leukaemia disorder release DAMPs S100A8/9 to induce mitochondrial dysfunction, oxidative stress, and DNA damages in HSCs via p53 activation in a paracrine manner and promote malignant transformation [95].

## 6. Inflammation and HSC Niche

**Figure 2:**
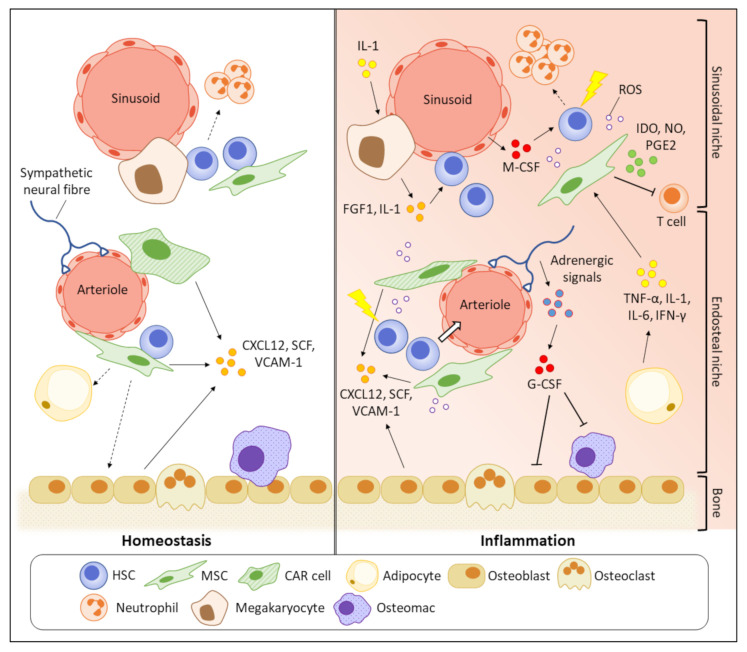
Pathogenic BM remodelling increases inflammatory stress on HSCs. In homeostasis, primitive HSCs prefer to reside in close proximity to sinusoids (sinusoidal niche) where the HSCs are supported by the HSC maintenance factors, such as CXCL12 and VCAM-1, secreted by various HSC niche cells. Inflammation suppresses the functions of HSC niche cells to produce the HSC maintenance factors, making the microenvironment unstable to support the HSC quiescence and contributing to HSC mobilisation. The HSC niche cells also participate in the secretion of inflammatory cytokines and impair the self-renewal potential of HSCs. The enriched pro-inflammatory cytokines and growth factors finally damage the primitive HSCs by promoting their cell cycling and apoptosis.

BM is a primary haematopoietic organ with high heterogeneous cellularity. Diverse non-hematopoietic cells such as osteogenic lineage cells, osteoclasts, endothelial cells, sympathetic nerves, and MSCs, together with haematopoietic cells, construct a stable bone structure to support the primitive phenotypes of HSCs [96]. The quiescent HSCs were believed to preferentially stay in close proximity to endosteum than central BM region, indicated by that most HSCs homed to the endosteal region after transplantation [97]. This, together with the HSC-supportive functions of osteoblasts, suggested that the endosteal niche might have a pivotal role in regulating the HSC quiescence [98]. However, it was subsequently found that such bias in spatial distribution towards endosteum is attributed to damages in BM vasculatures, particularly in sinusoids, after irradiation [99]. With advanced microscopic technology, it was later discovered that the majority of HSCs reside near sinusoidal blood vessels in central BM (sinusoidal niche), where they are supported by surrounding LepR^+^CXCL12^high^ MSCs [100]. Upon inflammation, the whole BM undergoes a pathological remodelling, making the microenvironment no longer stable to maintain the HSC function (Figure 2). Key cytokines/growth factors or signalling molecules secreted by the HSC niche cells for haematopoietic regulation under inflammatory conditions are summarised in Table 1.

***Osteoblasts:*** osteoblasts constitute the majority of bone cellular populations, and thus are one of the major contributors to the endosteal niche. It is reported that *Staphylococcus aureus* infection can suppress the osteoblasts and lead to bone loss and osteomyelitis by increasing G-CSF levels in the blood and BM [112]. G-CSF alters morphology and gene expressions of osteoblasts via modulating adrenergic signals from the sympathetic nervous system (SNS) [113,114]. Despite its suppression on osteoblasts, G-CSF fails to trigger HSC mobilisation under osteoblastic depletion, indicating that osteoblasts are indispensable to HSC mobilisation by collaborative function with other cell types [113]. It is recognised that the G-CSF-induced osteoblast suppression is indirectly mediated by osteoblast-supportive osteal macrophages (osteomacs), which are depleted in the G-CSF treatment [104]. The depletion of macrophages disrupts the endosteal niche and reduces the levels of HSC maintenance factors, such as CXCL12, Kit-ligand (KL), and angiopoietin-1 (Ang-1), to drive HSC mobilisation [104,106]. Moreover, considering that depletion of macrophages impairs erythroid recovery especially under myeloablative stress, it is suggested that apart from osteoblasts, osteomacs might act on megakaryocytes (MKs), which have close contact with the HSCs, to indirectly regulate the stem cells under inflammatory conditions [105,107]. While MKs maintain quiescence of HSCs by TGF-β1 secretion in homeostasis, MKs promote HSC expansion after myeloablative injury by FGF1 secretion [107]. Another study reported that depletion of CD169+ BM macrophages suppresses Nestin^+^ MSCs’ expression of HSC retention genes, including *Cxcl12*, *Kitl*, *Angpt1,* and *Vcam1*, and leads to HSC mobilisation [106].

***Sympathetic nervous system (SNS):*** BM is innervated by sympathetic nerve fibres and the expression of neurotransmitter receptors on immune cells allows the neuronal signals to affect the haematopoietic responses [120,121]. Lithium treatment which is used for treating bipolar disorders has been reported to promote neutrophilia and mobilisation of human HSPCs [115]. Adrenergic neurotransmitters can promote HSC proliferation, mobilisation, and repopulation potential via canonical Wnt signalling [120]. Upon altered circadian rhythms, which can be considered as non-inflammatory stress, the disrupted adrenaline signals dysregulate the CXCL12 expression by non-osteoblastic BM stromal cells, as well as the cyclic schedule of HSC mobilisation to the bloodstream [119]. Another study discovered that nociceptive nerves work with the sympathetic nerves and directly trigger HSC mobilisation by secreting calcitonin-gene-related peptides (CGRPs) that act on HSCs via receptor activity modifying protein-1 (RAMP1) and calcitonin-receptor-like receptor (CALCRL) [117]. Defects in nerve conduction in *Cgt*^−/−^ mice prevent G-CSF-induced HSC mobilisation despite the reduced CXCL12 level inside BM [118].

***Endothelial cell:*** as discussed, majority of HSCs are localised adjacent to blood vessels, indicating that BM vasculatures are important niches for HSCs to maintain their quiescence and differentiation [100]. The specific dependence of HSCs on the endothelial cells is also supported by the reduction in HSCs but not HPCs after conditional deletion of *Scf* from endothelial cells in perisinusoidal niche albeit unclear mechanisms [122]. Similar finding indicated that myeloid progenitors are specifically enriched around a subset of M-CSF expressing endothelial cells [103]. During infection, the reduced endothelial-derived M-CSF causes monocyte-dendritic cell progenitors (MDPs) to migrate away from the blood vessels and decreases their differentiation ability [103]. In a mouse model of chronic inflammation driven by endothelial MAPK activation, the impaired vascular integrity and HSC activities, such as survival, mobilisation and BM reconstitution are restored by NF-κB inhibition, which can suppress endothelial inflammation and alleviate cellular hypoxia levels and ROS generation [101]. Interestingly, there is a crosstalk between HSCs and endothelial cells that HSCs also participate in regulating inflammatory responses of the endothelial cells. BM vasculature expansion in IFNα-mediated acute inflammation is mediated by direct activation of endothelial VEGF signalling and indirect activation in haematopoietic cell-dependent manner [102].

***Mesenchymal stem cells (MSCs):*** MSCs are the other important multipotent precursor cells in BM with the ability to self-renew and differentiate to adipocytes, chondrocytes, osteoblasts, and myocytes. In other words, the majority of the non-haematopoietic HSC niche cells originate from MSCs. Nestin^+^ MSCs and LepR^+^ MSCs have close intact with HSCs and are highly enriched in HSC supportive cytokines such as CXCL12 and SCF [122,123]. They also promote the engraftment of transplanted HSCs probably due to their strong immunoregulatory effects in the HSC niche [124,125]. Upon inflammatory signals, MSCs massively secrete immunosuppressive molecules to inhibit the proliferation of T cells, B cells, and NK cells in BM [28,109]. Examples of immunosuppressive mediators include indoleamine 2,3-dioxygenase (IDO), prostaglandin E2 (PGE2), and nitric oxide (NO) [28,109]. It is been recently reported that mitochondria from MSCs can be transferred to CD4+ T cells to induce their differentiation to immunoregulatory Treg cells and restrict the inflammatory responses [126]. Another study revealed that viral LCMV infection and its associated IFN-γ signals impair HSC fitness by remodelling the structural network in the HSC niche and suppressing the function of CXCL12 abundant reticular (CAR) cells, a subset of MSCs, to secrete the HSC-supportive molecules [110].

## 7. Future Directions and Clinical Implications

During the past few decades, it has become clear that during inflammation, apart from haematopoietic progenitors, primitive HSCs also, directly and indirectly, receive the inflammatory insults despite the immunosuppressive HSC niche they reside in. HSCs are activated by PRRs or pro-inflammatory chemokines/cytokines to induce mobilisation, proliferation, and differentiation to replenish blood-immune systems for host defence. Emerging evidence shows that BM undergoes remodelling upon inflammatory stress, and the changes in the HSC niche are critical to escalate the haematopoietic responses. Although HSCs also show reciprocal impacts on HSC-niche cells, such as endothelial cells, to regulate their inflammatory responses [102], the crosstalk between the HSC niche cells and HSCs under inflammatory conditions is mostly unclear and still needs to be better dissected. Understanding of interplay between HSC and their niche provides lifelong support to maintain HSC homeostasis and immune system, prevention of inflammation-induced HSC exhaustion/malignant transformation.

Upon acute microbial insults, while transient haematopoietic responses are quickly triggered, haematopoietic cells develop a memory-like long-term epigenetic reprogramming for stronger innate immune responses during re-encounter with the pathogens. This newly emerging concept of innate immune memory is termed “trained immunity” [127]. The trained immunity has been first discovered in phagocyte lineages such as NK cells, monocytes, and macrophages. However, considering the short lifespan of monocytes, the innate immune memory is possibly inherited from myeloid progenitors or HSPCs. The establishment of trained immunity in HSPCs is supported by recent reports that HSPCs can be epigenetically reprogrammed by exposure to microbial challenges and improve resistance to secondary infection via enhanced myeloid differentiation [128,129]. Clinical evidence also showed that Bacillus Calmette–Guérin (BCG) vaccination can confer trained immunity by reprogramming human HSPCs [130]. The mechanism of trained immunity is currently still full of mysteries. Is innate immune memory pathogen specific? Are there any critical regulators that control the establishment of trained immunity? How to balance the infection-induced HSC damage and the acquisition of immune memory? Despite all these questions awaiting to be answered, the emergence of the trained immunity concept opens a new window to the existing vaccine strategies. We are convinced that future research on trained immunity in HSPCs can provide long-lasting immune protection to human populations, especially under the currently emerging global pandemics.

The current understanding of inflammation-induced haematopoietic responses has made the great achievement to introduce clinical use of cytokines such as G-CSF and GM-CSF for myeloid regeneration and CXCR4 antagonists for HSPC mobilisation. Immunoregulatory IFN-γ has been demonstrated to suppress the proliferation of leukaemia-initiating cells in acute lymphoblastic leukaemia (ALL) and AML [131,132]. Interestingly, while glycolysis keeps normal HSC and MSC quiescent, malignant stem cells rely heavily on mitochondrial oxidative respiration for energy production and maintenance of stem cell properties [133,134,135]. This unique metabolism in the malignant HSCs can be partially attributed to the mutations in mitochondrial isocitrate dehydrogenase 2 (IDH2) to induce abnormal glycolytic pathways in AML patients [133]. The intrinsic oxidative resistance in leukaemic stem cells and the extrinsic protection from the activated MSCs can also help the malignant cells to escape from the oxidative-stress-induced damages [136,137]. However, only 20% of the AML patients bear the *IDH1* or *IDH2* mutations [138]. Why can the majority of leukaemic stem cells gain higher competitive fitness under extensive oxidative stress? Do they switch their metabolism as a strategy to enhance their stem cell potential? How do normal HSCs change their metabolism during transformation? The current knowledge is still far from sufficient to answer these questions. A better understanding of the transition of energy metabolism may be beneficial for developing novel approaches to restore or prevent the loss of HSC quiescence during inflammation and the subsequent induced transformation process.

## Figures and Tables

**Table 1:** Summary of inflammatory responses of HSC niche cells under different inflammatory/stress conditions.

Inflammation/Stress		Cell Type	Regulators	Inflammatory Responses	Reference
Chronic	Obesity	Adipocyte	TNF-α IL-1β IL-6	Adipocytes secreted diverse inflammatory cytokines and accumulated adipocytes impaired reconstitution potentials, increased myelopoiesis, and suppressed lymphopoiesis of HSCs	[77,79,80,81]
Chronic	MAPK-induced inflammation	Endothelial cell	NF-κB	Impaired HSC survival and functionality	[101]
Acute	pI:C/IFN-α administration	Endothelial cell	VEGF	Vasculature expansion by haematopoietic and non-haematopoietic pathways	[102]
Acute	*Listeria monocytogenes* infection	Endothelial cell	M-CSF	Loss of endothelial-derived CSF1 disrupted localisation of myeloid progenitors in perisinusoidal niche and, in turn, promoted dendritic cell generation	[103]
Acute	G-CSF administration	Macrophage (Osteomac)	n.d.	G-CSF administration depleted BM macrophages, and in turn, suppressed HSC-supportive osteoblasts	[104]
Acute	Haemolytic anaemia	Macrophage (Osteomac)	n.d.	The presence of macrophages is critical to erythroid recovery	[105]
Acute	Macrophage depletion	Macrophage (Osteomac)	n.d.	Macrophage depletion suppresses MSCs’ expression of HSC retention genes	[106]
Acute	5-FU	Megakaryocyte (MK)	FGF1	MKs supported HSC regeneration by increasing FG1 secretion	[107]
Chronic	Obesity	Megakaryocyte (MK)	IL-1β	Obesity augmented MK and platelet function and upregulated their inflammatory gene expressions	[108]
Chronic	*Porphyromonas gingivalis* infection	Megakaryocyte (MK)	IL-1β	Increased platelet production	[108]
Acute	TNF-α, IFN-γ, IL-1α/β signals	Mesenchymal stem cell (MSC)	IDO NO PGE2	Activated MSCs secreted immunosuppressive molecules inhibited T cell proliferation and activities	[28,109]
Acute	LCMV infection	Mesenchymal stem cell (MSC)	IFN-γ	LCMV infection disrupted structural morphology, network, and capability of HSC-supportive cytokine secretion of CAR cells	[110]
Acute	NP-CGG immunisation	Neutrophil	n.d.	Neutrophil emigration from BM to create a vacancy in BM to promote myeloid cell generation	[111]
Acute	*Staphylococcus aureus* infection	Osteoblast	G-CSF	Osteoblastic suppression by G-CSF impaired osteoblasts’ support to HSCs and promoted HSC mobilisation	[112,113,114]
Acute	Lithium treatment	Sympathetic nerve	β-catenin	Increased HSPC proliferation, mobilisation, and granulocyte colony formation	[115]
Acute	Adrenergic neurotransmitter treatment	Sympathetic nerve	β-catenin	Increased hCD34+ HSPC proliferation, mobilisation, and repopulating potential in vivo via canonical Wnt signalling pathway	[116]
Chronic	Neurotransmission ablation	Sympathetic nerve	G-CSF	Neurotransmission ablation suppressed HSC mobilisation and osteoblast function	[117,118]
Chronic	Altered circadian rhythms	Sympathetic nerve	CXCL12	Altered adrenergic signals disrupted rhythmic CXCL12 oscillations in BM and in turn dysregulation circadian HSC mobilisation	[119]
Acute	Allograft transplant	Treg cells	IL-10	The presence of Treg cells was critical to support the survival of allo-HSCs	[2]

n.d., not determined.

## Data Availability

Not applicable.
